# Bending Performance of Alkali-Activated Concrete Beams Based on Digital Image Correlation Method

**DOI:** 10.3390/ma18071616

**Published:** 2025-04-02

**Authors:** Hongbo Gao, Hongna Sun, Zhaokun Wang, Xiaoyan Han, Xinru Li

**Affiliations:** 1 School of Civil Engineering and Architecture, Hainan University, Haikou 570228, China; gaohongbo@hainanu.edu.cn (H.G.); sunhongna@hainanu.edu.cn (H.S.); 2 School of Hydraulic and Civil Engineering, Ludong University, Yantai 264025, China; wzk3440274458@163.com; 3 State Key Laboratory of Coastal and Offshore Engineering, Dalian University of Technology, Dalian 116024, China; hxy12006040@mail.dlut.edu.cn

**Keywords:** alkali-activated concrete, four-point bending test, digital image correlation method, crack width

## Abstract

To optimize the design of concrete beams and assess the performance of new materials, four-point bending tests were conducted to examine the bending behavior of alkali-activated concrete (ALAC) beams with different reinforcement ratios. The cracking load, displacement, and crack width were measured using the Digital Image Correlation (DIC) method and compared with results obtained through traditional methods. The findings demonstrate that DIC significantly outperforms traditional techniques in determining crack load, displacement, and crack width, particularly in capturing crack initiation and propagation. As the reinforcement ratio increases, the mid-span displacement at the peak load decreases for ALAC beams. At the same reinforcement ratio, the ALAC beam exhibits 1.07 times the bearing capacity, 1.4 times the mid-span displacement, and 0.72 times the maximum crack width compared to ordinary Portland cement concrete (PCC) beams. The cracking load, calculated using a plasticity coefficient of 1.17 for the section resistance moment, aligns closely with the experimental results. Furthermore, the formulas for mid-span displacement and maximum crack width in ordinary concrete beams can also predict the corresponding properties of ALAC beams. These findings validate the mechanical behavior and application potential of ALAC beams under various reinforcement ratios.

## 1. Introduction

Alkali-activated concrete (ALAC) is an innovative cementitious material created by activating industrial by-products such as blast furnace slag, fly ash, and metakaolin with an alkaline activator [1,2,3]. ALAC has gained considerable attention due to its substantial reduction in carbon dioxide emissions during production, with emissions being 50% to 80% lower than those of conventional Portland cement concrete [4,5,6]. Additionally, ALAC exhibits rapid hardening, high strength, and exceptional resistance to high temperatures, freezing, corrosion, and permeability [7,8,9].

Although ALAC has garnered considerable attention in both academia and industry for its potential to replace traditional Portland cement concrete (PCC), its slow development of early strength, particularly under normal temperature curing conditions, has hindered its widespread application. Thomas et al. [10] highlighted that, while alkali-activated concrete based on fly ash exhibits excellent corrosion resistance, high strength, and low dry shrinkage, it typically requires high-temperature curing to fully leverage these advantages. Nath et al. [11] proposed that the incorporation of a small amount of Portland cement into alkali-activated concrete could enhance its early strength under normal curing conditions. Yang Cheng et al. [12] further validated that the mechanical properties of alkali-activated concrete mixed with a small proportion of cement at normal temperature are comparable to those of alkali-activated concrete without cement reinforcement under high-temperature curing conditions. In addition, Bezemer et al. [13] reported that the long-term mechanical properties of ACLC deteriorated, which was different from PCC and should be given sufficient attention.

In practical engineering applications, flexural members play a crucial role in concrete structures. Traditional measurement methods, such as visual observation, displacement meter placement, and strain gauge installation, are not only time-consuming but also provide limited, single-point data, which restrict the measuring effectiveness [14]. Digital Image Correlation (DIC), a high-precision, non-contact, full-field deformation measurement technique, has seen significant development and widespread application in recent years [15,16,17]. Initially proposed by Yamaguchi [18] and Peters et al. [19], the DIC method has evolved continuously, enabling its use in the study of deformation and failure in concrete beams. Yan et al. [20] introduced a method for global fracture identification and reconstruction using DIC, overcoming the limitations of traditional techniques. Xiong et al. [21] employed the DIC method to capture the entire crack development process in reinforced concrete beams. Pohoryles et al. [22] validated the accuracy of DIC in measuring strain and displacement through tests on composite reinforced concrete beams. Baietti et al. [23] used the DIC method to estimate the size of the fracture process zone of alkali-activated mortars with different aggregate types and particle sizes. However, to date, no research has explored the application of the DIC method in the flexural performance testing of ALAC beams.

In response to this gap, this study aims to conduct four-point bending tests on ALAC beams with different reinforcement ratios, utilizing DIC to determine the cracking load, displacement, and crack width. Additionally, the cracking load, mid-span displacement, and maximum crack width will be predicted using existing calculation formulas, with the predicted values compared to the experimental results.

## 2. Experimental Programs

### 2.1. Specimen Preparations

The coarse aggregates used in this study consist of gravel with a particle size range of 5 to 20 mm, while the fine aggregates are medium sand. Grade S95 mineral powder produced by BORUN Company in Zhengzhou of China is selected, and its density is 2.9 g/cm^3^. The fly ash used is secondary fly ash, with a density of 2.23 g/cm^3^. The composition of fly ash is divided into 56% SiO_2_, 26% Al_2_O_3_, 9% Fe_2_O_3_, 6% CaO, and 3% MgO. The cement employed is locally produced P.O 42.5 ordinary Portland cement from Hainan. According to the information provided by the manufacturer, the cement is composed of 82% cement clinker, 15% fly ash, and 3% CaSO_4_, of which the cement clinker contains 59% C_3_S, 21% C_2_S, 10% C_4_AF, 7% C_3_A, 2% CaO, and 1% MgO. The alkaline activator comprises 98% pure solid NaOH and the Na_2_SiO_3_ solution with a modulus of 2.3 (13.5% Na_2_O, 30% SiO_2_, and 56.5% H_2_O). NaOH and Na_2_SO_3_ are mixed according to the mass ratio of 11:84, and the molar concentration of the alkaline solution is 6.33M. The polycarboxylic acid-based superplasticizer is also used. The concrete mix proportions are detailed in Table 1.

**Figure 1:**
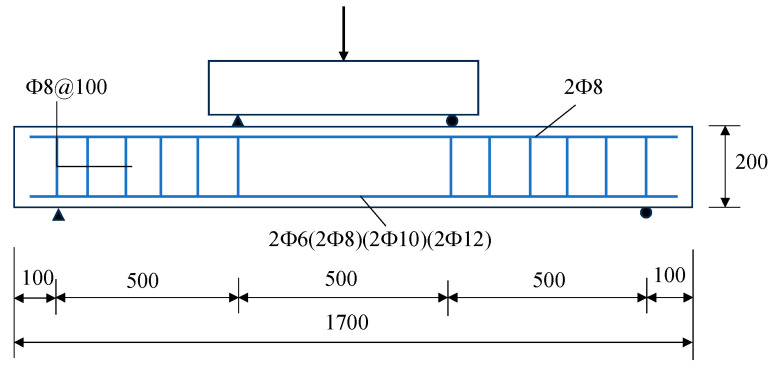
Dimensions and reinforcement of beams (unit: mm).

A total of five beams were designed, including 4 ALAC beams and 1 PCC beam. The cross-section of each beam is 200 mm × 120 mm, with a total length of 1700 mm and a span of 1500 mm. Each beam is reinforced with two longitudinal reinforcements on both the tension and compression sides. Two 8 mm diameter steel reinforcements were placed in the compression zone. Based on the reinforcement ratio recommended by Chinese national standards [24], two 6 mm, 8 mm, 10 mm, and 12 mm diameter steel reinforcements were used in the tension zone to investigate the effect of different reinforcement ratios in the tension zone on the bending performance of ALAC beams. For comparison, two 12 mm diameter steel reinforcements were installed in the tension zone of the PCC beam. All reinforcements are HRB400 grade. All beams and control specimens were cured in a natural environment with an average temperature of 32 degrees Celsius and an average humidity of 80%. After 28 days of curing, the material mechanical properties of the control specimens were tested and are listed in Table 2, in which each mechanical parameter is determined by the average test value of 6 control specimens. The specific dimensions and reinforcement layout of the test beams are shown in Figure 1, with the reinforcement ratios of each test beam provided in Table 3.

### 2.2. Four-Point Bending Tests

**Figure 2:**
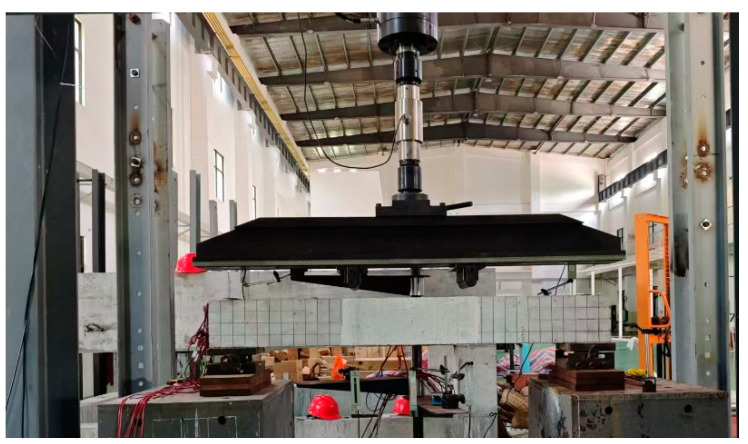
Testing device.

The four-point bending test was employed in this study, as illustrated in Figure 2. A displacement meter is set at the mid-span position to measure the mid-span displacement, and a displacement meter is set at the support to eliminate the production displacement. Prior to the test, a thin layer of lime water was applied to one side of the beam surface. After the water dried, a 50 mm × 50 mm square grid was drawn to facilitate the measurement of crack width and monitor crack development using a reading microscope. A speckle pattern was applied on the other side of the beam, that is, the surface of the beam was first covered with white spray paint, and black paint spots were evenly sprayed after the paint dried to provide reference points for the DIC analysis. The specimen information monitored by the DIC method included crack growth, cracking load, and displacement, and the frequency of photo acquisition was 1 Hz.

**Figure 3:**
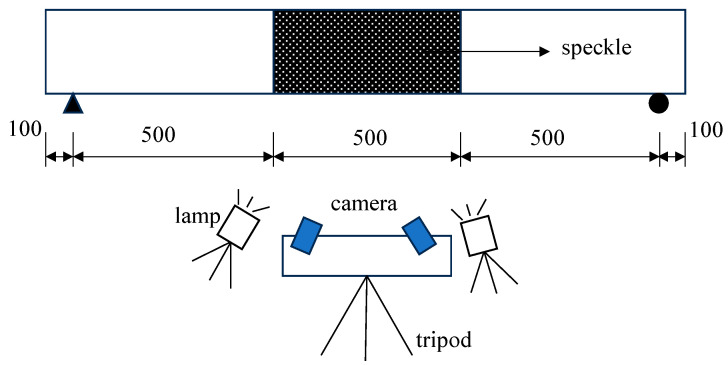
DIC setup diagram (unit: mm).

The test was conducted using a 500 kN hydraulic testing machine manufactured by WANCE Company in Shenzhen, China. During the initial stage, a stepped loading method was employed, with each loading increment set at 5 kN. Once the load reached 90% of the calculated ultimate load, the loading mode switched to displacement control until the test beam failed. The layout of the test setup is depicted in Figure 2. The DIC method was carried out using two cameras, which required fine-tuning and calibration prior to the test. Given the potentially prolonged loading process, two LED fill lights were used to ensure adequate image quality. To maintain equipment stability throughout the test, the cameras and fill lights were mounted on a tripod for precise positioning. After data collection, the recorded data were analyzed and processed using VIC-3D software (Version 9.4.40), by which the rigid body displacement can be automatically removed. The specific setup of the DIC system is shown in Figure 3.

### 2.3. Failure Mode and Crack Propagation of Beams

**Figure 4:**
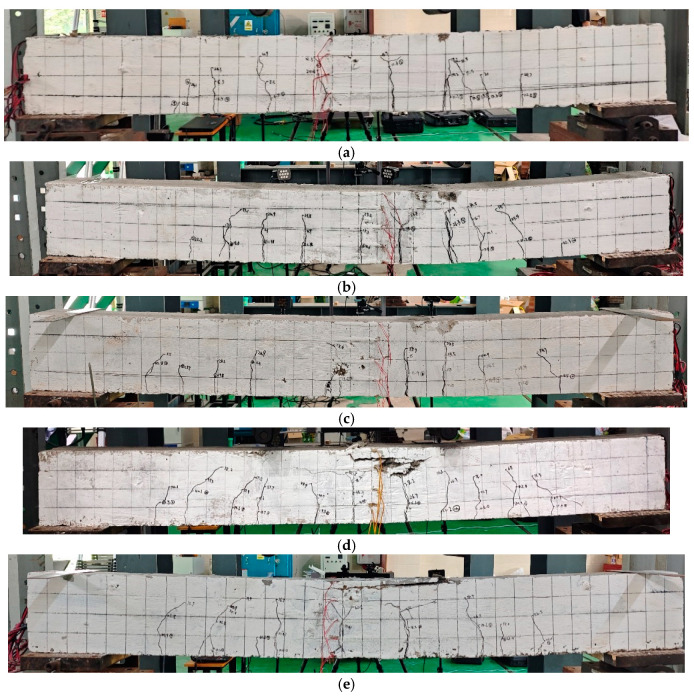
Failure pattern of beams. (**a**) A-6. (**b**) A-8. (**c**) A-10. (**d**) A-12. (**e**) P-12.

The A-6 beam exhibited low-reinforcement failure during the test. Specifically, during the loading process, the reinforcement in the tension zone reached its yield strength and fractured, while the concrete in the compression zone remained uncrushed, as shown in Figure 4a. This type of failure typically occurs suddenly and without warning, posing a significant risk to structural safety. In contrast, the other beams experienced proper reinforcement failure. After concrete cracking, the cracks gradually expanded and developed at a relatively slow rate, accompanied by the formation of new, smaller cracks. As the load continued to increase, the displacement of the beam also increased. When the tensile reinforcement reached its yield point, the load increase slowed, but the displacement of the beam increased rapidly until the concrete in the compression zone was ultimately crushed, as shown in Figure 4b–e. Figure 4a–e show the crack distribution and propagation of the specimen at failure.

**Figure 5:**
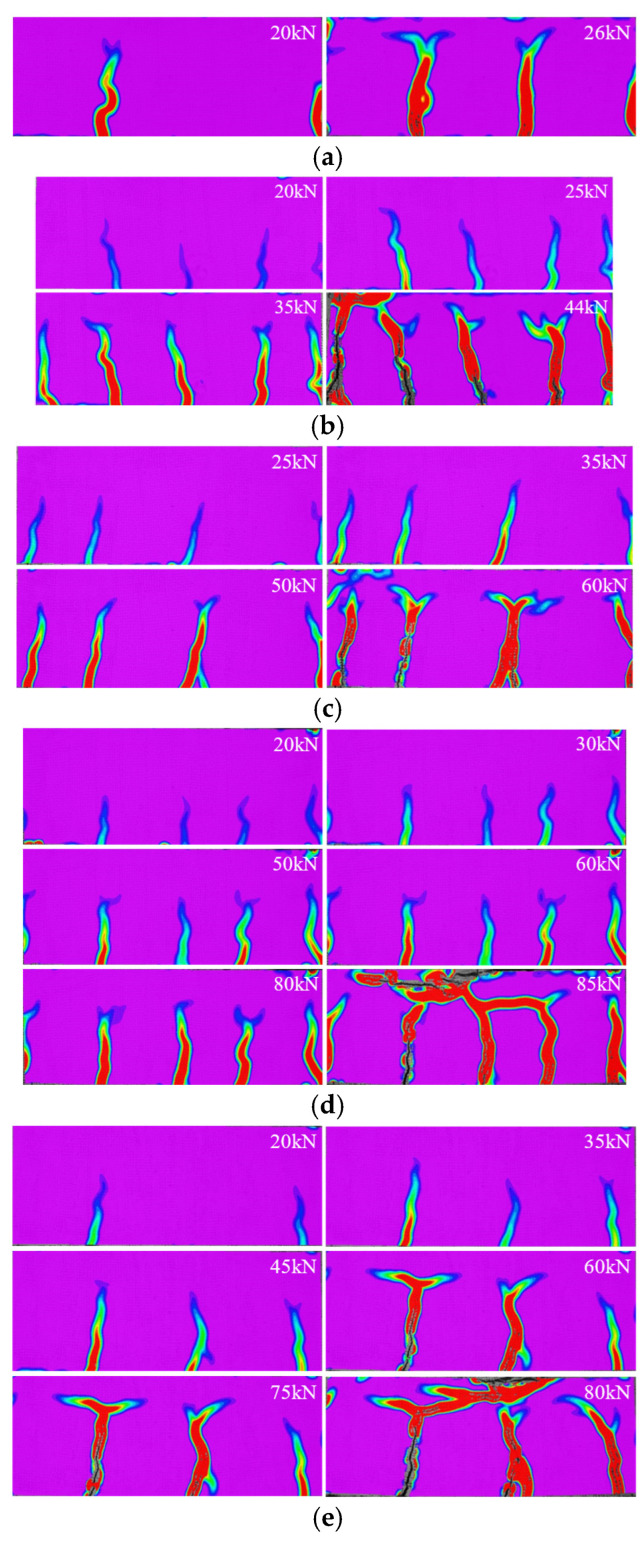
Crack distributions. (**a**) A-6. (**b**) A-8. (**c**) A-10. (**d**) A-12. (**e**) P-12.

To observe the crack development in greater detail, the DIC method was employed to capture the progression of cracks. It was found that for beam A-6 that suffered low-reinforcement failure, the number of cracks was small, and the width was large, as shown in Figure 5a. For the remaining beams that suffered proper reinforcement failure, ALAC beams showed a larger number of cracks and a smaller crack width compared to PCC beams, as shown in Figure 5b–e. Furthermore, as the load increased beyond a certain point, the cracks in beams stopped propagating upwards, with the widths continuing to expand.

## 3. Result Analysis

### 3.1. Cracking Load

In this study, two methods were used to determine the cracking load of the beams, including the traditional visual observation method and the DIC technique. In the DIC method, after removing the influence of environmental noise, the crack is considered to develop when the strain is greater than the ratio of the tensile strength and elastic modulus of the concrete (*f*_t_/*E*_c_). Table 4 presents a comparison of the cracking loads of beams determined by the different methods, and the correlation coefficient is 0.67.

The data indicate that the cracking load determined by the DIC method is generally lower than that obtained through traditional visual observation. Conventional visual observation relies on the moment when the observer identifies the first visible crack, which typically occurs after the crack has propagated to a certain extent. As a result, this method may record a higher value than the actual cracking load. In contrast, the DIC method provides detailed information on the strain field of the concrete surface, capturing early-stage changes associated with the formation of small cracks, thus allowing for a more accurate determination of the actual cracking load. In conclusion, while traditional visual observation methods are simple and direct, accuracy is constrained by the ability of observers to detect cracks. The DIC method, with its high precision and non-contact nature, offers a more reliable assessment of cracking load, enabling a more accurate understanding of material behavior and structural properties.

### 3.2. Load-Displacement Curve

**Figure 6:**
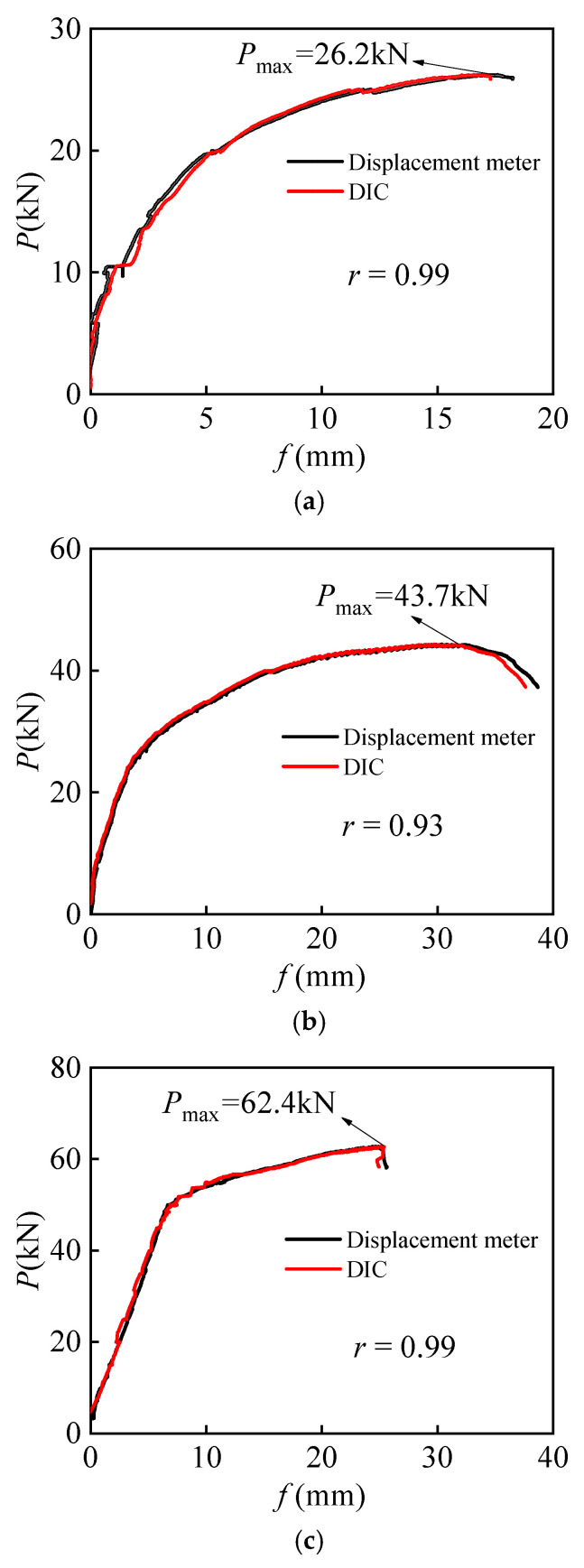

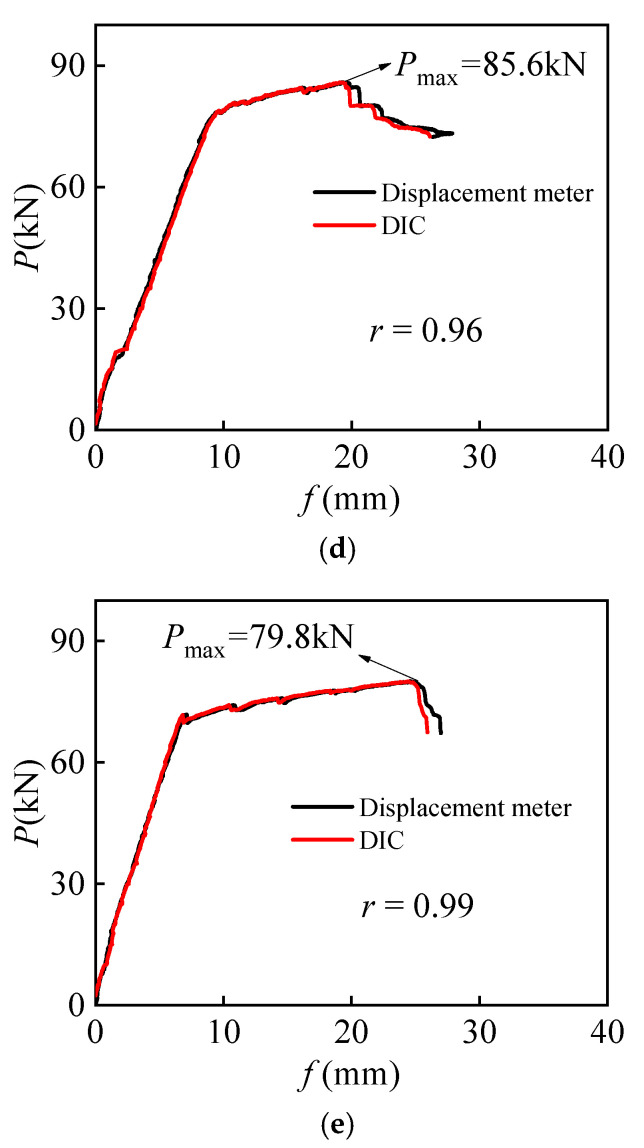
*P*-*f* curves of beams. (**a**) A-6. (**b**) A-8. (**c**) A-10. (**d**) A-12. (**e**) P-12.

The load-mid-span displacement (*P*-*f*) curves of ALAC beams with different reinforcement ratios are shown in Figure 6a–d, and the corresponding curves of PCC beams are shown in Figure 6e. As observed from the figure, the load-displacement curves of most beams exhibit a distinct yield plateau. At this stage, although the load increases slowly, the displacement increases sharply, resulting in a clear yield plateau, indicating the good ductility of structures. In contrast, the A-6 beam does not show an obvious yield plateau in the load-displacement curve due to the failure of a few reinforcement steels. The failure of this beam is brittle, as it reached its load capacity and failed rapidly before the reinforcement could yield.

For ALAC beams with different reinforcement ratios, the mid-span displacement corresponding to the peak load decreases as the reinforcement ratio increases. This is because a higher reinforcement ratio leads to greater flexural stiffness of the section post-cracking, thus enhancing the bearing capacity and reducing the corresponding displacement. Moreover, for beams with the same reinforcement ratio, the ALAC beams exhibit higher ultimate bearing capacity than the PCC beams. The load-displacement curve results obtained through the DIC method closely match those measured by traditional displacement meters, demonstrating that the DIC method can accurately capture the displacement of reinforced concrete beams while avoiding the potential errors and inconveniences associated with displacement meter installation.

### 3.3. Load-Crack Width Curve

**Figure 7:**
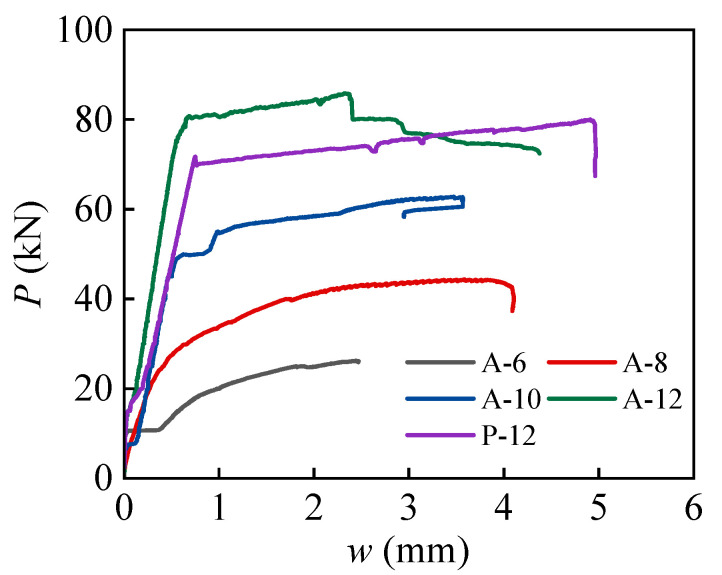
*P*-*w* curves of beams.

Using the virtual extensometer in the VIC-3D software, the cracks in the pure bending zone of beams were accurately monitored. The main crack width was obtained and correlated with the load data from the loading device to generate the load-crack width (*P*-*w*) curve, as shown in Figure 7. The experimental results demonstrate a significant difference in the maximum crack width between ALAC beams and PCC beams under the same load conditions. Specifically, as the reinforcement ratio decreases, the crack width increases, indicating that the crack width is highly sensitive to the reinforcement ratio. For the A-12 and P-12 beams, which have the same reinforcement ratio but differ in cementing material, it was observed that the maximum crack width of the A-12 beam is consistently smaller than that of the P-12 beam. This suggests that ALAC beams exhibit slower crack propagation under bending load compared to PCC beams.

## 4. Comparison Between Theoretical Calculation and Experimental Values

### 4.1. Cracking Moment

**Figure 8:**
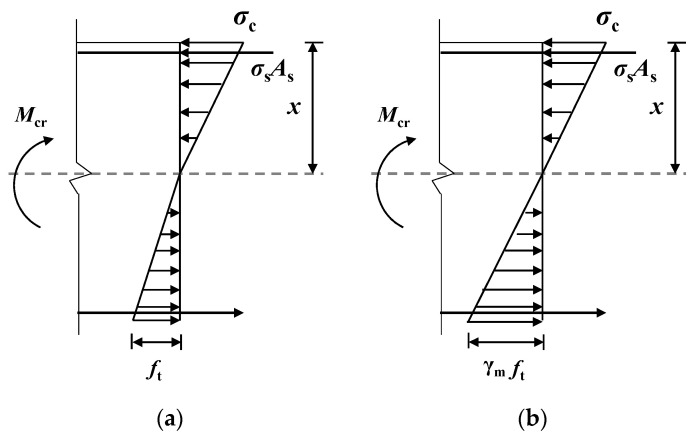
Cracking moment calculation diagram. (**a**) Simplified stress distribution diagram. (**b**) Calculated stress distribution diagram.

At the moment the reinforced concrete beam is about to crack, the tensile strain at the edge of the tensile zone reaches the ultimate tensile strain *ε*_tu_ of the concrete. The stress distribution in the tensile zone exhibits a curved pattern with distinct plastic characteristics, and the maximum tensile stress reaches the tensile strength *f*_t_ of the concrete. In contrast, the concrete in the compression zone remains in a nearly elastic state, with the stress distribution approximating a triangular shape. The sectional strain follows the assumption of the plane section, as illustrated in Figure 8a. Under the condition of equal cracking moment, Figure 8a can be simplified to Figure 8b, where the stress distribution in the tension region is represented as a straight-line pattern.

At this point, the edge stress in the tension region transitions from *f*_t_ to *γ*_m_*f*_t_, where *γ*_m_ is the resistance moment plasticity coefficient of the cross-section. The cracking moment *M*_cr_ of the beam is given by:

(1) $$M_{\mathrm{cr}}=\gamma_{m}f_{t}W_{0}$$

where *W*_0_ is the resistance moment of the section and ft is the axial tensile strength of concrete. From the above formula, it can be observed that the value of *γ*_m_ is crucial for calculating the cracking load, which is derived as follows.

As shown in Figure 8b, by neglecting the influence of reinforcement steel in the compression zone, the balance of forces can be expressed as:

(2) $$\frac{1}{2}\sigma_{c}bx=\frac{1}{2}f_{t}b(h-x)$$

Then,

(3) $$\sigma_{c}bx=f_{t}b(h-x)$$

where *b* and *h* are the width and height of the section, respectively. Based on the assumption of the plane section, the compressive strain of the concrete can be expressed as:

(4) $$\varepsilon_{c}=\frac{x}{h-x}\varepsilon_{\mathrm{tu}}$$

When concrete is about to crack, due to the development of plastic deformation in the tensile zone, the ratio of tensile strain to compressive strain is no longer governed by the elastic modulus *E*_c_, but by the deformation modulus, which can be approximated as 0.5*E*_c_. Therefore:

(5) $$\varepsilon_{\mathrm{tu}}=\frac{2f_{t}}{E_{c}}$$

(6) $$\sigma_{c}=\varepsilon_{c}E_{c}=\frac{2f_{t}x}{h-x}$$

By substituting Equation (6) into Equation (3), we obtain:

(7) $$x=\frac{h}{\sqrt{2}+1}=0.414h$$

According to the torque balance, we can derive:

(8) $$M_{\mathrm{cr}}=\frac{1}{2}\sigma_{c}bx\times \frac{2}{3}x+\frac{1}{2}f_{t}b(h-x)\times \frac{2}{3}(h-x)$$

Combining Equations (1) and (8), and simplifying, then:

(9) $$\gamma_{m}=\frac{M_{\mathrm{cr}}}{f_{t}W_{0}}=\frac{0.195f_{t}bh^{2}}{0.167f_{t}bh^{2}}=1.17$$

Therefore, for a rectangular-section ALAC beam, the plastic coefficient of the section resistance moment, *γ*_m_, is 1.17. The cracking moment of the beam is calculated using Equation (1). The calculated cracking bending moment and the test values, obtained using the DIC method, are presented in Table 5. It can be observed that the predicted values derived from the formula align closely with the experimental values with a correlation coefficient of 0.95.

### 4.2. Mid-Span Displacement

According to the Chinese National Standard GB 50010-2010 [24], under normal service state, the formula for calculating the displacement of reinforced concrete beams is:

(10) $$f=\frac{Pa}{48B}\left(3{l_{0}}^{2}-4a^{2}\right)$$

The flexural stiffness *B* of the section of the flexural member is given by:

(11) $$B=\frac{M_{k}}{M_{q}\left(\theta -1\right)+M_{k}}\cdot B_{s}$$

The short-term stiffness *B*_s_ of the flexural member is given by:

(12) $$B_{s}=\frac{E_{s}A_{s}{h_{0}}^{2}}{1.15\psi +0.2+\frac{6\alpha_{E}\rho }{1+3.5\gamma_{f}}}$$

The strain non-uniformity coefficient *ψ* of longitudinal reinforcement between cracks is defined as:

(13) $$\psi =1.1-\frac{0.65f_{\mathrm{tk}}}{\rho_{\mathrm{te}}\sigma_{s}}$$

where, *E*_s_ is the elastic modulus of the steel reinforcement, taken as 200 GPa. *f*_tk_ is the axial compressive strength of concrete. *ρ*_te_ is the effective longitudinal tensile reinforcement ratio. *σ*_s_ is the longitudinal reinforcement stress. *ρ* is the longitudinal tensile reinforcement ratio. *γ*_f_ is the compression flange reinforcement factor. *M*_k_ and *M*_q_ are the bending moments calculated according to the load standard combination and quasi-permanent combination, respectively. *θ* is the coefficient considering the effect of long-term load on the increase in displacement. *P* is the load value applied to the component. *a* is the shear and bending segment length. *l*_0_ is the calculation span of beams.

Table 6 presents the comparison between the measured and calculated displacement values at the mid-span of the beam under the 0.6 ultimate load condition. For the beams that suffered proper reinforcement failure, the correlation coefficient between the experimental results by the DIC method and calculated results is 0.93.

The data indicate that the displacement predictions based on short-term stiffness align well with the experimental values. Furthermore, the displacement of ALAC beams is generally higher than those of PCC beams. For the same reinforcement ratio, the displacement of the ALAC beams is approximately 1.40 times that of the PCC beams. This finding suggests that under identical conditions, ALAC beams may exhibit greater deformation capacity, potentially due to differences in material properties or internal microstructure.

### 4.3. Crack Width Calculation

According to the Chinese National Standard GB 50010-2010 [24], the formula for calculating the maximum crack width *w*_max_ of the reinforced concrete beam is:

(14) $$w_{\max}=\alpha_{\mathrm{cr}}\psi \frac{\sigma_{s}}{E_{s}}\left(1.9c_{s}+0.08\frac{d_{\mathrm{eq}}}{\rho_{\mathrm{te}}}\right)$$

where *α*_cr_ is the characteristic coefficient of stress. *c*_s_ is the concrete cover thickness. *d*_eq_ is the equivalent diameter of the longitudinal tensile reinforcement.

The maximum crack width predicted under the 0.6 ultimate load, calculated using Equation (14), and the experimental values obtained through the DIC method are shown in Table 7. For the beams that suffered proper reinforcement failure, their correlation coefficient is −0.74.

It is found that the calculated values show good agreement with the experimental data for the beams that suffered proper reinforcement failure. This suggests that the crack width under normal service state can be reliably predicted using Equation (14). Furthermore, for ALAC beams, the maximum crack width decreases as the reinforcement ratio increases. Additionally, ALAC beams exhibit smaller maximum crack widths compared to PCC beams, even with lower reinforcement ratios.

## 5. Conclusions

This study performed bending tests on ALAC beams with different reinforcement ratios and employed the DIC method to obtain the cracking load, displacement, and crack width. Furthermore, calculation formulas were used to predict the cracking load, mid-span displacement, and maximum crack width under normal service state, and the predicted results were compared with the experimental results. The key conclusions are as follows:

1. The DIC method outperforms traditional measurement techniques in assessing cracking load, displacement, and crack width. It offers precise, non-contact measurements of full-field deformations, enabling accurate determination of the cracking load and continuous tracking of crack propagation under different load conditions;
2. For ALAC beams with different reinforcement ratios, the mid-span displacement corresponding to the peak load decreases as the reinforcement ratio increases, indicating that a higher reinforcement ratio enhances the stiffness of structures and reduces displacement. Furthermore, for the same reinforcement ratio, ALAC beams demonstrate higher ultimate bearing capacity and smaller maximum crack width compared to ordinary PCC beams, highlighting the superior mechanical properties of ALAC;
3. For ALAC beams, the cracking load calculated using a plasticity coefficient of 1.17 for the section resistance moment aligns well with the experimental results. Additionally, the calculation formulas for mid-span displacement and maximum crack width, which are typically used for ordinary concrete beams, also provide accurate predictions for the corresponding properties of ALAC beams under normal service state. This indicates that existing design codes and calculation methods can, to some extent, be applied to ALAC, offering valuable theoretical support for engineering application.

This study verified the reliability and engineering application potential of ALAC and demonstrated the advantages of the DIC method in structural crack observation. On this basis, two future research directions are provided. Firstly, ALAC materials can be used not only for structures but also for functional concrete, such as Autoclaved Aerated Concrete [25], which has good thermal insulation and sound insulation properties. Secondly, the DIC method can be used for crack monitoring of materials with uncertain crack propagation directions, such as fiber-reinforced concrete, UHPC, and ECC. However, it is worth noting that due to the small strain value corresponding to the development and extension of cracks, DIC method is susceptible to the influence of ambient noise, especially for research that requires long-term observation [26].

## Figures and Tables

**Table 1:** Mix proportion of concrete (kg∙m^−3^).

	Gravel	Sand	Mineral Powder	Fly Ash	Cement	NaOH	Na_2_SiO_3_	Water	Superplasticizer
ALAC	1047	690	250	85	85	11	84	155	10
PCC	1047	690	-	70	350	-	-	190	10

**Table 2:** Mechanical parameters of materials.

Material	Cube Compressive Strength *f*_cu_ (MPa)	Axial Compressive Strength *f*_tk_ (MPa)	Axial Tensile Strength *f*_t_ (MPa)	Elastic Modulus *E*_c_ (GPa)
ALAC	53.4	41.1	3.10	29.7
PCC	54.6	32.5	3.14	34.5

**Table 3:** Reinforcement information of beams.

Specimen	Type of Concrete	Diameter of Reinforcement (mm)	Ratio of Reinforcement (%)
A-6	ALAC	6	0.29
A-8	ALAC	8	0.51
A-10	ALAC	10	0.81
A-12	ALAC	12	1.17
P-12	PCC	12	1.17

**Table 4:** Cracking load of beams.

Specimen	Traditional Visual Observation Method (kN)	DIC Method (kN)
A-6	12.1	10.32
A-8	13.6	10.90
A-10	11.5	10.91
A-12	14.0	12.04
P-12	13.5	12.37

**Table 5:** Measured and calculated cracking moments.

Specimen	Measured Cracking Moment (DIC) (kN·m)	Calculated Cracking Moment (kN·m)	Ratio of Measured to Calculated Cracking Moment
A-6	2.58	2.93	0.88
A-8	2.73	2.94	0.93
A-10	2.73	2.95	0.93
A-12	3.01	2.96	1.02
P-12	3.09	2.98	1.04

**Table 6:** Measured and calculated displacement.

Specimen	Measured Displacement (DIC) (mm)	Calculated Displacement (mm)	Ratio of Measured to Calculated Displacement
A-6	3.21	2.11	1.52
A-8	4.11	3.57	1.15
A-10	4.70	4.06	1.16
A-12	6.02	4.55	1.32
P-12	4.31	4.02	1.07

**Table 7:** Measured and calculated crack width.

Specimen	Measured Crack Width (DIC) (mm)	Calculated Crack Width (mm)	Ratio of Measured to Calculated Crack Width
A-6	0.61	0.17	3.59
A-8	0.46	0.35	1.31
A-10	0.40	0.36	1.11
A-12	0.36	0.35	1.03
P-12	0.50	0.32	1.56

## Data Availability

The original contributions presented in this study are included in the article. Further inquiries can be directed to the corresponding author.
